# Hypogonadism in the Aging Male Diagnosis, Potential Benefits, and Risks of Testosterone Replacement Therapy

**DOI:** 10.1155/2012/625434

**Published:** 2012-03-14

**Authors:** Prasanth N. Surampudi, Christina Wang, Ronald Swerdloff

**Affiliations:** Division of Endocrinology, Department of Medicine, Harbor UCLA Medical Center and Los Angeles Biomedical Institute, Torrance, CA 90509, USA

## Abstract

Hypogonadism in older men is a syndrome characterized by low serum testosterone levels and clinical symptoms often seen in hypogonadal men of younger age. These symptoms include decreased libido, erectile dysfunction, decreased vitality, decreased muscle mass, increased adiposity, depressed mood, osteopenia, and osteoporosis. Hypogonadism is a common disorder in aging men with a significant percentage of men over 60 years of age having serum testosterone levels below the lower limits of young male adults. There are a variety of testosterone formulations available for treatment of hypogonadism. Data from many small studies indicate that testosterone therapy offers several potential benefits to older hypogonadal men. A large multicenter NIH supported double blind, placebo controlled study is ongoing, and this study should greatly enhance the information available on efficacy and side effects of treatment. While safety data is available across many age groups, there are still unresolved concerns associated with testosterone therapy. We have reviewed the diagnostic methods as well as benefits and risks of testosterone replacement therapy for hypogonadism in aging men.

## 1. Introduction

 Hypogonadism in older men is a syndrome characterized by the presence of low testosterone levels and clinical signs and symptoms of hypogonadism. The symptoms of hypogonadism can include decreased libido, impaired erectile function, muscle weakness, increased adiposity, depressed mood, and decreased vitality. Hypogonadism is more common in aging men and is is also referred to as late-onset hypogonadism (LOH) [1], androgen deficiency in the aging male (ADAM) [2], partial androgen deficiency in the aging male (PADAM) [3], testosterone deficiency syndrome (TDS) [4], and andropause [5]. We prefer LOH over the other descriptors.

 A significant percentage of men over 60 years of age have serum testosterone levels below the lower limits of young male adults (20 to 30 years) [6–9]. One longitudinal study has suggested that approximately 20% of men in their 60s and approximately 50% of men in their 80s have serum total testosterone (TT) levels significantly below those of the levels of normal young men [8]. European Male Aging Study (EMAS) estimated much lower prevalence (2.1%) of symptomatic late-onset hypogonadism in the in the population [10]. Several other studies have also noted a decline in TT with age [11]. In some instances, the clinical symptoms/manifestations are more difficult to recognize because they may be masked by comorbid illnesses. There has been some controversy as to the significance of falling testosterone levels with age. Most experts believe that it is a medically significant condition resulting in significant detriment to the quality of life and adversely affecting the function of multiple organ systems [6, 11]; while others suggest that it is a chemical marker of generalized illness [11].

 Over the past two decades, significant advances have been made in improving the understanding of the pathophysiology of the hypogonadism, the diagnostic methods used to diagnose low testosterone levels, and testosterone replacement therapy. In spite of these advances, a great deal of confusion and misunderstanding still exists among clinicians and patients about diagnosis of hypogonadism in aging men, and benefits and risks associated with testosterone therapy. In this paper, we have reviewed the studies reported in the literature on this subject and attempted to address the important questions pertaining to hypogonadism in older men. (1) How to diagnose LOH in aging males? (2) What are the best treatment options available today for clinicians to treat LOH? (3) Will older hypogonadal men benefit from testosterone treatment? (4) What are the risks associated with such an intervention?

## 2. Prevalence of Hypogonadism in Aging Males

 Several longitudinal and cross sectional studies have been carried out to determine the prevalence of hypogonadism in men. Some of the important cross-sectional and longitudinal studies reported include Baltimore Longitudinal Study of Aging (BLSA), Boston Area Community Health Survey (BACHS, European Male Aging Study (EMAS), and Massachusetts Male Aging Study (MMAS) [8–10, 12]. These studies have reported different prevalence rates of hypogonadism in men. The differences may be in part due to different definitions of hypogonadism adopted by these studies. Variables include low testosterone level definition, clinical symptoms used for the diagnosis of hypogonadism, the population studied, and the inclusion or exclusion of comorbid conditions in older men.

The actual prevalence of low-serum testosterone in aging men is not known with certainty, but it is projected to be up to 25% [8, 10, 13, 14]. The EMAS noted an overall prevalence of hypogonadism of 2.1% [10]. The study noted an increase with age from 0.1% for 40- to 49-year-old men to 5.1% for 70- to 79-year old men. The MMAS reported that the overall prevalence of symptomatic androgen deficiency was 5.6% with an increased prevalence of 18.4% among 70-year-old men. In the Boston Area Community Health Survey, the overall prevalence of hypogonadism was 5.6% among older men of age 30–79, and the survey also suggested that the prevalence among 70-year-old men could be 18.4% [9]. In a study of men in Hong Kong, the prevalence of symptomatic hypogonadism was 9.5% with an increased prevalence of 16.7% in the older age group (60–64 yrs) [15]. The BLSA reported that 19% of men over 60 years had low testosterone levels with the average decline of TT was 3.2 ng/dL per year among men who had an average age of 53 years at entry into BSLA [8].

A summary of these studies is given in Table 1. Some of the important findings of these studies are (1) prevalence hypogonadism in men (based on symptoms and/or total testosterone levels) increases with age starting from the fourth decade, and (2) the hypogonadism was projected to be much higher in aging men with comorbidities such as metabolic syndrome, type 2 diabetes (T2DM), and cardiovascular disease (CVD). Some other studies have pointed out that much of the increase in prevalence of hypogonadism with age can be ascribed to comorbid conditions [16–18].

## 3. Causes

 Hypogonadism can be classified as primary, secondary, and mixed hypogonadism. Primary Hypogonadism results from disorders of the testes that lead to low testosterone production and impaired fertility. The laboratory values for patients with primary hypogonadism show low testosterone and elevated LH and FSH levels. Secondary hypogonadism results from disorders of the hypothalamus and the pituitary. The laboratory values for men with secondary hypogonadism show low testosterone and low or inappropriately normal LH and FSH levels. Mixed hypogonadism can result from dual defects in the testes and in the pituitary-hypothalamic axis. The laboratory values for mixed hypogonadism can be varied including cases with low testosterone with mild increases in LH and FSH levels.

 Often the type of hypogonadism in older men is either secondary or mixed hypogonadism. The decline in testosterone levels can be due to several factors including (1) decline in Leydig cell function, (2) decline in pituitaryhypothalamic axis function with loss of circadian variation (3) increase in the levels of SHBG, (4) changes in testosterone receptors sensitivity, and (5) effects of altered cardiometabolic and inflammatory markers [20–22].

Most aging males do not have congenital etiology as their cause hypogonadism. These congenital processes would show up much earlier than later in life. Aging males are more likely to have an acquired cause or idiopathic etiology of hypogonadism. The diagnosis of hypogonadism in aging men requires the clinician to evaluate for other causes of secondary and mixed hypogonadism such as hypothalamic-pituitary disease, hyperprolactinemia, depression, chronic alcoholism, diabetes mellitus, and infiltrative diseases such as hemochromatosis and medications (e.g., opioids, anabolic steroids, and glucocorticosteroid, opioid analgesics, antidepressants cimetidine, spironolactone, and antifungal drugs).

## 4. Diagnosis of Hypogonadism in Aging Males

**Figure 1:**
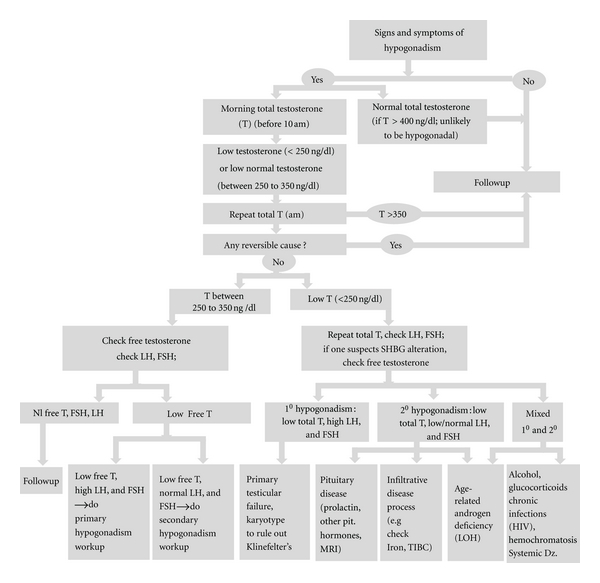
Algorithm for the diagnosis of hypogonadism in aging males.

 Questionnaires have been developed to help identify aging males with hypogonadism. These questionnaires include (1) Androgen Deficiency in Aging Male (ADAM) questionnaire, (2) Aging Male survey (AMS), and (3) MMAS questionnaire [23, 24]. While these questionnaires can have high sensitivity, they have low specificity [25]. Because of their low specificity, most guidelines do not recommend their use. A new questionnaire has recently been validated for hypogonadal men and its usefulness is not yet established in older men [26]. Testosterone deficiency should be confirmed by laboratory measurements for older men who are identified as hypogonadal by means of these questionnaires [23, 24]. The Endocrine Society Consensus Committee recommends that the diagnosis of hypogonadism should be based on identification of symptoms and signs suggestive of testosterone deficiency and presence of low testosterone levels measured by a reliable assay on two or more occasions [27]. The algorithm suggested for the diagnosis of hypogonadism in aging males described in Figure 1.

 The symptoms and signs of hypogonadism in aging men vary depending upon the age, severity and duration of androgen deficiency, comorbid illnesses, androgen sensitivity, and previous testosterone therapy [28]. Symptoms and signs suggestive of hypogonadism (Table 2) include loss of vitality, visceral obesity, decreased muscle mass and strength, osteopenia and bone pain, and mood changes and depression [1, 2, 5]. Other nonspecific symptoms are decreased energy, motivation, and initiative, poor concentration and memory, sleep disturbance, increased sleepiness, increased body fat, and diminished physical or work capacity [1, 2, 5, 10].

 The measurement of the serum testosterone concentration is usually the most important single diagnostic test for male hypogonadism. The important factors that need to be considered in testosterone measurement are (1) types/forms of testosterone to be measured, (2) time of measurement, and (3) frequency of measurement. 

Three different forms of testosterone can be measured and they include (a) total testosterone level, (b) free or unbound testosterone, and (c) bioavailable testosterone. About 30% to 50% of testosterone is bound to albumin with low affinity. The free testosterone is about 1% to 2%. The bioavailable fractions of testosterone are composed of both the albumin-bound and the free testosterone. The rest of the testosterone is bound to sex hormone-binding globulin (SHBG), and this portion is not available for use by most target organs [21, 27–29].

Measurement of the serum testosterone concentration is dependent on the time of measurement. In healthy males, the circadian rhythm affects GnRH secretion and causes testosterone levels to change throughout the day. Testosterone levels are highest in the morning and start to decline by 10 am. The lowest value of testosterone is observed approximately by 10 PM [30]. The variability between morning and evening testosterone levels decreases in older men because of changes to the circadian rhythm. Testosterone levels should be measured in the morning to obtain peak testosterone results. If a single morning value is low or borderline low or does not fit with the clinical findings, the measurement should be repeated once or twice before making the diagnosis of hypogonadism.

 The normal reference range for TT in adult men is approximately 300–1000 ng/dL. If the early morning serum TT level is less than 250 ng/dL, the patient is likely to be hypogonadal. A repeat TT measurement is required to confirm the diagnosis. Further evaluation is required if the TT is in the grey zone of 250 to 350 ng/dL. The follow-up tests required include a repeat of TT levels. One should evaluate the FT levels when TT values are in the grey zone. If the results indicate a low TT and/or low FT levels, then the patient is hypogonadal. These labs then should be followed with testing of the serum gonadotrophins (LH, FSH) levels to help ascertain the anatomical level of hypogonadism (e.g., primary testicular or hypothalamic/pituitary). If a TT level is <150 ng/dL or there are signs or symptoms of possible mass lesion in the pituitary, then one should order pituitary imaging to exclude pituitary and/or hypothalamic tumor or infiltrative disease. The diagnosis of hypogonadism in aging men should never be undertaken during an acute illness as it can result in temporarily low testosterone levels [27, 31].

 TT levels can be measured directly by automated immunoassays and immunometric assay methods [32–35]. However, there is a growing concern about the accuracy of automated immunometric assays especially for measurements in the low testosterone concentration range [36–38]. Currently, the most accurate method for determining the TT to differentiate eugonadal from hypogonadal males is liquid chromatography-tandem mass spectrometry [38]. Bioavailable testosterone is measured by the ammonium sulfate precipitation method. The equilibrium dialysis is the reference method for the measurement of free testosterone concentrations [28]. This latter method is very complex and tedious and thus a routine measurement is only available in reference laboratories. Free and bioavailable testosterone concentrations can also be estimated (calculated) from TT and SHBG concentrations. The calculated FT concentrations derived from using the law of mass action equations provide a convenient and reasonable alternative to equilibrium dialysis methods [39–41]. The calculations of FT are limited by assumptions made for the equilibrium dissociation constants (Kd) for the binding of SHBG and T and albumin and T. In addition, there is no agreed standard for determining the SHBG. The tracer analog displacement assays that are available in many hospital laboratories are inaccurate and their use is not recommended [31].

## 5. Treatment of Hypogonadism with Testosterone

 There must be a definitive diagnosis of hypogonadism before the treatment is initiated. Borderline testosterone levels alone are not necessarily an indication to begin testosterone replacement therapy. There must be a combination of signs, symptoms and issues with patient's quality of life [27, 42, 43]. The goal of testosterone therapy is to raise serum testosterone level into the midnormal range (400–700 ng/dL) and resolution or reduction in symptoms of hypogonadism. However, the ultimate goals of therapy are to reduce disease and disability, maintain or improve quality of life, and hopefully add vitality to the years [44].

The Endocrinology Society Guidelines recommend that the health care provider considers avoiding testosterone replacement therapy in men with certain conditions. The guidelines do not recommend testosterone replacement therapy for those who still desire fertility [27]. Aging males with a history of severe lower urinary tract obstruction, untreated sleep apnea, prostate cancer, or breast cancer should not be considered for testosterone replacement therapy [27]. Individuals with an abnormal digital rectal examination suggestive of cancer, and/or elevated prostate-specific antigen should have a careful evaluation by an urologist before considering treatment. An elevated hematocrit (e.g., >50%) is also considered to be a relative contraindication for testosterone replacement therapy [27]. Other contraindications include poorly controlled heart failure and an American Urological Association International Prostate Symptom Score (IPSS) >19. Testosterone replacement therapy can be considered, for individuals that are treated with medications to decrease the urinary obstructive symptoms of BPH or congestive heart failure.

 There are several types of testosterone preparations that are currently available in the United States including testosterone injections, scrotal and nonscrotal transdermal patches, oral testosterone, buccal testosterone, and testosterone gel preparations. Advantages and disadvantages associated with different formulations are given in Table 3. Currently, testosterone injections and testosterone gel preparations are more commonly used in the United States. Medications that stimulate the production of endogenous testosterone (i.e., hCG, clomiphene) may be used in the treatment of older men when fertility is an issue. 

The buccal testosterone preparation had several drawbacks including a fixed dose of 30 mg and problems with adhesion of the tablets to the gums [45]. There are men who have not been able to tolerate the feeling of local presence of the buccal testosterone tablet. In the United States, an oral testosterone is currently not in use because preparations of oral 17-alkylated androgens (e.g., methyltestosterone and fluoxymesterone) have an increased risk for hepatic toxicity and abnormal lipid profile (elevated LDL, low HDL) [46]. Oral testosterone undecanoate (TU) is available in many parts of the world but is not approved for use in the United States [46, 47]. The presently available oral TU has a short pharmacokinetic profile and must be taken with food. It leads to variable serum testosterone levels, elevated DHT/T ratio, and variable clinical response [46, 47].

 There are several intramuscular injectable preparations available including testosterone enanthate, testosterone cypionate, and TU. Testosterone enanthate or testosterone cypionate injections can be administered with 200 mg every 2 weeks [48–50]. The peak levels of testosterone can be achieved within 2-3 days after administration of testosterone enanthate and cypionate. Serum testosterone levels have to be measured midway between injections, in individuals that are on biweekly testosterone enanthate or cypionate injections. Adjustments to the administration of testosterone dosage have to be made when T is > 700 ng/dL or T < 350 ng/dL [27, 48–51]. The long acting TU injection has been approved in Europe but it is not yet approved in the United States. It can be administered in Europe as 1000 mg injection with a loading dose, followed by another injection of TU 1000 mg at week 6, and subsequently administered at every 12 to 14 weeks [51]. The serum testosterone levels rise to supraphysiologic levels for several days and gradually decline over a period of 10 to 14 weeks after administration of TU injection [27, 51–53]. The high peak levels can be avoided by administering 750 mg instead of 1000 mg of TU injections [54]. The levels can be checked prior to each subsequent injection. The TU injection requires a large volume and can cause cough in a small number of cases [27, 51–53]. The testosterone levels from the longer acting TU cannot be reduced quickly if PSA levels start to rise. This can pose a risk for an aging male.

 The transdermal method of testosterone administration has been used as scrotal and nonscrotal patches. Scrotal patches are not currently used because of the need to shave or cut scrotal hair to maintain adequate patch adhesion to the skin [51, 55–57]. In addition, some individuals complained of scrotal itching or discomfort. Transdermal patches can deliver 5 to 10 mg of testosterone. The levels of TT should be checked 3–12 h after application of the patch, and dose adjustments should be made to achieve testosterone level in the midnormal range [51, 55–57]. While nonscrotal patch can help achieve normal serum testosterone levels with a diurnal variation, skin irritation can occur in up to 30% of patients [55–57]. Other drawbacks include the need for up to two patches per day in some men. A testosterone-in-adhesive matrix patch is also available. It can deliver approximately 4.8 mg of T/d and last for 2 days. Like other transdermal patches, it can cause some skin irritation [58].

 Most testosterone gels are hydroalcoholic-based gels and contain 1-2% testosterone. The testosterone is absorbed into the skin and is slowly released into the body. It allows for a fairly steady level of serum testosterone that is as effective as the patch. Testosterone gel is packaged as sachet, metered pump, or underarm testosterone-gel preparation. The most widely used testosterone gel can come as 2.5 g, 5 g, 7.5 g, or 10 g gel with the usual dose being between 5–10 g per day. One application of the gel contains 50–100 mg of testosterone. It is intended to deliver approximately 5 to 10 mg testosterone to the body which can match the normal production of testosterone in healthy men. The dose adjustment of testosterone gel can be done after a patient has been treated for at least one week to achieve serum testosterone level in the midnormal range. Testosterone gel has minimal skin irritability. However, they is a potential of transfer of testosterone to others upon close skin contact. Transfer of testosterone can cause clinical virilization in females and children, and this can be minimized by showering or wearing clothing.

Of the transdermal testosterone preparations, the gel formulation is currently recommended for most hypogonadal men. The gel formulation is able to produce a steady serum testosterone concentration within the physiological range of adult men. Some men who desire freedom from daily application or lower cost can use intramuscular injections of testosterone. Oral testosterone formulations that do not have side effects of prior oral drugs are under development.

 Aging males who are started on testosterone replacement therapy should be followed periodically. After the initiation of testosterone replacement therapy, subjects should have a clinic visit, within 3 months, to make needed dosage and formulation adjustments. Subjects should have regular visits (3 to 6 months after treatment initiation and then annually) for assessment of symptom improvement [27]. The visits should also focus on evaluating for erythrocytosis, prostate disease, difficulties with sleep apnea, and other adverse events. The urinary frequency or voiding difficulties can be affected by prostate size. The International Prostate Symptom Scale (IPSS) can be used to monitor for changes by using it at baseline and subsequent visits. An IPSS prostate symptom score of >19 should warrant a urological consultation [27]. In addition to TT levels, lab tests should include measures of liver function, hemoglobin, hematocrit, and PSA. A hematocrit is to be evaluated at baseline, 3 to 6 months, and then annually. When hematocrit rises above 54%, one should cease testosterone replacement therapy until hematocrit decreases to a safe level before reinitiating therapy at a lower dose as noted in the recent Endocrine Society Guidelines [27]. The patient with significantly elevated hematocrit should be monitored and evaluated for symptoms of sleep apnea, cardiovascular events, and hypoxic complaints.

## 6. Possible Benefits of Testosterone Therapy

 In the past two decades, several studies have been carried out to determine the benefits of testosterone replacement therapy for hypogonadal men. The results of these studies indicate that testosterone therapy provides several benefits including improvements in muscle mass and strength, bone mineral density (BMD), adiposity, lipid abnormalities, glucose control, cardiovascular disorders, sexual function, mood, and cognitive function. A summary of the benefits of testosterone therapy for aging hypogonadal males is given below.

### 6.1. Muscle Mass and Strength

In aging males, falling testosterone levels have been associated with declining strength and muscle mass [59, 60]. The New Mexico Aging Process Study also noted correlations between total and free testosterone levels and muscle strength [19, 61]. A cross sectional study of 118 men on androgen deprivation therapy for prostate cancer showed impaired physical and functional musculoskeletal performance when they were compared with age-matched controls who were not hypogonadal [62].

 Several studies found testosterone replacement therapy to be beneficial in improving muscle strength in hypogonadal older men. Svartberg et al. noted an improvement in hand grip in older men with TU treatment [63]. Page et al. found improvements in both hand grip and physical function for hypogonadal men that were on testosterone enanthate treatment [64]. A modest increase in muscle mass and an improved leg extensor strength were also observed in some studies [65, 66]. A double-blinded placebo-controlled study found that elderly men on testosterone replacement therapy for 6 months improved their lower limb muscle strength (isometric knee extension peak torque) when compared with subjects on placebo [67]. The improvement in muscle strength was accompanied by other measurable benefits in gait and balance, aggregate locomotor function test, physical performance test, and 6min walk test at 6-month assessment in the testosterone group [67]. However, the improvements in muscle strength did not result in significant changes in functional ability. Meta-analyses of randomized trials in middle-aged and older men have confirmed that testosterone administration is associated with an increase in lean body mass (LBM), reduction in fat mass, and increase in grip strength when compared with placebo.

Some other studies did not observe any significant improvements in muscle strength with testosterone therapy. A small study by Clague et al. found no significant improvements in handgrip strength, isometric strength of knee flexors, and extensors or leg extensor power [68]. Meta analysis studies by Isidori et al. also did not find any significant improvement in muscle strength when middle-aged men were treated with testosterone replacement therapy [69]. Similar results were reported by Nair et al. with testosterone patch replacement study in elderly men [70].

 Physical function is affected by many factors including muscle strength. Improvements in physical function have also been studied. Changes in performance-based measures of physical function have been inconsistent across testosterone trials that recruited healthy older men. Testosterone therapy did not significantly affect overall quality of life scores [27, 71]. However, a Spanish study confirmed that physical function and the ability to participate in physical activity safely are related to feelings of well being in the elderly [72]. The long-term benefit of testosterone on functional improvements requires further investigation.

### 6.2. Bone

Testosterone plays an important role in BMD by increasing osteoblastic activity and reducing osteoclastic activity [73–75]. Some of the androgen effects on bone are partially indirect. The reduction in osteoclastic activity appears to be mediated via testosterone's aromatization product to estradiol (e.g., effects on cortical bone) [73–78]. There appears to be associations between testosterone levels and 25(OH) Vitamin D levels and testosterone and phosphate levels [79, 80]. There is a strong association between low bone density, bone loss, osteoporosis, and low testosterone levels in aging males [81, 82]. There has also been an association of increased risk of fractures in men with hypogonadal states [83–85].

Testosterone replacement therapy was found to increase bone density in hypogonadal men [63, 86]. The bone density increases, however, it may not reach normal adult bone mass [87]. Meta-analysis studies have shown testosterone replacement therapy positively affects bone density and reduces the rate of bone loss [69, 88]. Testosterone therapy appears to have a positive effect on bone markers with a reduction in bone resorption markers [24, 69]. While a few studies have not shown a clear benefit in bone density, many studies with exogenous testosterone have noted increases in BMD in hypogonadal aging males [89, 90]. The improvement in BMD was noted in most types of exogenous testosterone administration including the more commonly used gel preparations [70, 91].

Some studies have reported improvements in lumbar bone density [63, 91–93]. This has also been noted in the meta-analysis by Tracz et al. [88] and Isidori et al. [69]. The studies by Wang et al. [91], Amory et al. [90] and Nair et al. [70] suggested improvements in hip BMD while the meta-analysis studies by Tracz et al [88] and Isidori et al. [69] found femoral neck improvements to be inconclusive. While BMD improves, the effect of testosterone replacement therapy on fracture risk is still unclear. None of the studies have been large enough to show a fracture risk reduction with testosterone replacement therapy. Further, investigations are required to confirm the long-term benefits of testosterone therapy for improving bone strength and its properties.

### 6.3. Adiposity, Lipid Abnormalities, and Glucose Metabolism

Men with obesity, metabolic syndrome, and type-2 diabetes have low total and free testosterone and low sex hormone-binding globulin (SHBG). Low testosterone is associated with dyslipidemia, hypertension, obesity, and diabetes, all of which increase the risk of cardiovascular disease [93, 94]. It appears that testosterone levels are involved with obesity in a complex relationship. Testosterones levels could be as a causal factor of obesity and could also be a consequence of excess adipose tissue itself. A meta-analysis of observational studies noted that males with metabolic syndrome had lower TT and free testosterone levels [94].

Obesity has effects on the hypothalamus, pituitary gland, and testes with resultant hormonal abnormalities [95]. An increase in regional adiposity appears to be related to levels of low testosterone [96]. This can be associated with lower TT and sex hormone-binding globulin in obese individuals when compared with nonobese individuals [10, 95]. It appears that there is a negative correlation between serum TT and FT levels and visceral fat mass [97, 98].

Several studies have noted that testosterone replacement therapy results in a reduction of body fat mass and waist circumference in hypogonadal men with and without obesity [99, 100]. The studies have also noted some positive changes in total body fat and regional fat distribution with testosterone replacement therapy [95, 100–102]. Adiposity was also noted to decrease along with an increase in lean body mass in a muscle function trial of hypogonadal men in the testosterone-treated group [67]. BMI improved in only one trial [103] and body fat decreased in other studies [104, 105]. It has also been noted that the leptin levels correlate with body fat content and leptin levels decrease with testosterone replacement in T2DM and metabolic syndrome [104, 106]. Several studies indicate a decrease in central adiposity in men with metabolic syndrome and/or T2DM with testosterone treatment [103, 104, 107].

Testosterone plays a role in lipid metabolism. It does affect the actions of lipoprotein lipase and lipolysis [108]. Low testosterone levels can lead to changes in triglycerides and high-density lipoprotein cholesterol. It can also affect total cholesterol levels [69]. The effect of testosterone on lipid profile was investigated in several studies including those on coronary heart disease, metabolic syndrome, and diabetes [100]. Testosterone therapy results in a small but significant fall in total cholesterol and in some LDL cholesterol [100, 105, 107]. In one study, it was noted that the use of testosterone showed a dose-dependent trend toward lower HDL, LDL, and total cholesterol [109]. Most reports found no change in triglycerides. However, one meta-analysis noted that the testosterone and placebo/nonintervention groups did not differ significantly in the changes from baseline in total cholesterol, low-density lipoprotein (LDL), and triglycerides [110]. The observed decrease in LDL in a number of studies may be of some benefit to individuals with hypogonadism and other cardiovascular risk factors. In general, the effects on lipids are observed more after oral-and higher-dose testosterone treatment. The effects are less with transdermal preparation and lower doses of replacement.

 Hypogonadism with low total or free testosterone correlates with low HDL cholesterol [111]. The MMAS study noted a strong, positive relationship between HDL and testosterone in men with CVD [111]. However, treatment with testosterone does not seem to increase HDL. Some studies have observed that supraphysiologic doses of testosterone will lower HDL [110, 112]. High-density lipoprotein (HDL) cholesterol levels were also found to decrease in patients that were on oral testosterone therapy [57, 113]. In a meta-analysis study, the high-density lipoprotein (HDL) cholesterol levels were significantly lower in the testosterone-treated group than the control group [110]. There is some evidence that after an initial decrease, HDL cholesterol levels then return to baseline [105]. It is concerning to note that any cardiovascular benefit in lowering LDL is tempered by undesired changes to HDL levels.

There have been studies that have noted associations among hypogonadism, insulin resistance, T2DM, and metabolic syndrome. Low testosterone concentrations have been noted in individuals with T2DM [17, 114]. This was also noted in a review of the NHANES database [115]. While individuals with T2DM did not appear to have linear correlations between testosterone concentration and degree of glucose control, studies with testosterone treatment in hypogonadal men have shown some reductions in glucose levels and insulin resistance [103, 107, 114, 116]. Wang et al. has stated that the mechanisms that connect hypogonadism, insulin resistance, and T2DM are complicated and include inflammatory markers, oxidative stress, and many other possible underlying causes [117]. A mechanistic paradigm that may be involved between testosterone, obesity, and T2DM has been reviewed by Wang et al. [117].

 The effects of testosterone on insulin sensitivity and glucose control have been noted in studies. Low TT or SHBG levels are associated with T2DM [118, 119]. Marin et al. reported that testosterone improved insulin sensitivity assessed by euglycemic insulin clamp studies in obese men while reducing central adiposity [99]. A randomized double blind crossover trial demonstrated a significant reduction in insulin resistance in hypogonadal men with T2DM on testosterone replacement therapy [107]. Several other studies have also shown that testosterone therapy improves insulin sensitivity in hypogonadal men with and without T2DM [117]. Another study has noted improvements in insulin sensitivity once testosterone levels were normalized [120]. In one study, healthy men who had induced hypogonadism had a reduction in insulin sensitivity when there was an acute withdrawal of testosterone [121]. A small longitudinal study of TU versus placebo noted improvement in fasting glucose and decreased insulin resistance in the TU group [122]. The improvements in insulin sensitivity and glucose control are corroborated in other studies which also noted improvements in HOMA when hypogonadal men with metabolic syndrome and/or T2DM were treated with testosterone replacement therapy [70–72]. A prospective trial, by Jones etal., reported that hypogonadal men with metabolic syndrome and/or T2DM on transdermal TRT had reductions in HOMA-IR in addition to beneficial effects on total and LDL-cholesterol and lipoprotein-a [105]. Improvements in hemoglobin A1C (HbA1C is a glycated hemoglobin that reflects average plasma glucose concentration over several weeks) were also observed in two trials [103, 107]. A meta-analysis study has also noted improvements in fasting plasma glucose, and HOMA in the testosterone-replacement therapy group [118]. These studies suggest the normalizing testosterone levels may be helpful in individuals who have T2DM and hypogonadism.

### 6.4. Cardiovascular Disease

High levels of testosterone do not contribute to the etiology of cardiovascular disease and increased incidence of coronary atherosclerosis in men undergoing coronary angiography [123, 124]. However, cardiovascular risk factors may be affected by the presence of low testosterone levels. Low testosterone is associated with dyslipidemia, hypertension, obesity, and diabetes, all of which increase the risk of CVD [125, 126]. Lower testosterone levels were associated with adverse changes to carotid intima medial thickness and ankle/brachial index as a measure of peripheral arterial disease and calcific aortic atheroma [127–129].

Some observational studies show a correlation between low testosterone and CVD, and others show no correlation. Several epidemiological studies have noted that low testosterone levels were associated with increased mortality in older men [130–135]. In contrast, some studies such as the MMAS study did not show that TT levels were clearly related to all-cause mortality [136, 137].

 Aging males are at particular risk for CVD. There are studies that testosterone therapy may be beneficial in several ways. English et al. were the first to report that testosterone-replacement therapy may be beneficial for men with cardiac disease [138]. They found that 22 men with chronic stable angina who were treated with transdermal testosterone-replacement therapy had greater angina-free exercise tolerance than 24 placebo-treated controls. TRT administration in hypogonadal men was reported to improve exercise tolerance (decreased exercise-associated ischemia) in aging males with coronary artery disease [139]. A small longitudinal study of TU versus placebo noted improvements in the carotid medial thickness in the TU group [122]. Testosterone treatment was also found to decrease lipoprotein—A levels in subjects with metabolic syndrome and or T2DM [105, 140]. Another randomized control study showed testosterone treatment in elderly patients with chronic heart failure improved various cardio, respiratory, and muscular outcomes [94, 141].

 A meta-analysis study showed no significant differences in the rates of death, myocardial infarction, revascularization procedures, or cardiac arrhythmias between the testosterone and the placebo/nonintervention groups [110]. Another meta-analysis also found no association between testosterone replacement therapy and cardiac events [142]. However, these meta-analyses trials of testosterone therapy generally have not been designed or adequately powered to detect effects on clinically significant cardiovascular events [143]. Other studies of testosterone-replacement therapy have not demonstrated an increased incidence of cardiovascular disease or events such as myocardial infarction, stroke, or angina [144]. However, the true benefits of normalizing testosterone levels in aging hypogonadal men who have underlying cardiac disease are not fully understood and require further investigation.

### 6.5. Sexual Function

 When plasma testosterone levels are below a minimum level, many aging men experience symptoms of low libido, changes in erectile function, and possibly changes in morning erection frequency [145]. Low testosterone levels can lead to reduced sperm production (oligospermia), decreased libido and sexual satisfaction, and strength of erections in elderly men. In hypogonadal younger males, the restoration of serum testosterone levels to the normal levels show benefits on sexual function outcomes [146]. There are reports of improvements in sleep-related erections, cavernous venous leakage, and enhancing production of nitric oxide synthase following testosterone replacement therapy [147–149]. Many studies in younger men have reported improvements in libido in the testosterone-treated groups when compared with placebo groups [144]. While improvements in libido appear to be more consistent, the improvements to erectile function appear to be varied among trials [150–152]. The meta-analysis study by Isidori et al. reported that moderate improvement in sexual function was noted in men with testosterone levels below 346 ng/dL [152]. The overall impact on sexual satisfaction, however, appears to be unclear as to degree of improvement after testosterone replacement therapy [151]. Long term studies are required to further evaluate the effects of testosterone-replacement therapy on erectile function in older men.

### 6.6. Mood

 Aging hypogonadal men are at increased risk for developing depression [153]. In hypogonadal men, testosterone replacement was associated with improved mood and feelings of wellbeing [44, 154–156]. Despite improvements in mood with testosterone-replacement-gel therapy, the beneficial effect from concomitant testosterone-replacement therapy and SSRIs cannot be clearly differentiated [157, 158]. A meta analysis showed some beneficial effects of testosterone replacement therapy on depression scores [159]. Testosterone replacement therapy's beneficial effects on quality of life and depressive mood have not been consistent across trials [138, 154]. Meta-analysis studies and reviews of androgen replacement therapy do not appear to support testosterone replacement as an antidepressant for the general population [160, 161]. Additional studies are needed to assess the effects of testosterone on clinical depression.

### 6.7. Cognitive Function

The impact of testosterone on aspects cognitive function has been studied in trials [162–164]. Lower levels of testosterone appear to have an effect on abilities such as spatial abilities, verbal abilities, and cognitive function [162–166]. This is of particular importance to the aging male who may experience changes in cognitive ability from other comorbidities such as vascular disease and neurological pathology.

The effects of testosterone replacement therapy on measures of cognitive function and memory have shown mixed results [167, 168]. In studies by Janowsky et al., Cherrier et al., and Tan and Pu, testosterone replacement improved verbal and spatial memory and constructional abilities in nonhypogonadal men with mild cognitive impairment, hypogonadal men and Alzheimer's disease [165, 166, 169–171]. Transdermal testosterone treatment (5 to 10 mg of testosterone) of men aged 34 to 70 years appears to improve their verbal memory and spatial memory [170, 171]. Another study of healthy men aged 50 to 90 years investigated the efficacy of intramuscular testosterone (alone or in combination with the aromatase inhibitor anastrozole) in improving the cognitive function. This study noted improvements in spatial memory in both groups but verbal memory only improved in testosterone-treated men without anastrozole [171]. The beneficial effects on cognition, memory, and visuospatial abilities were not seen in other randomized studies [172–175]. The evidence in support of and against the improvements in cognition, memory, and visuospatial abilities is not uniform. This could be in part due to the short duration and smaller sample size. Randomized control trials of a longer duration are needed particularly in older hypogonadal men who are on testosterone replacement therapy to fully ascertain the benefits on cognitive performance.

## 7. Possible Risks of Testosterone Therapy for Elderly Men

Testosterone replacement therapy should be utilized with full awareness of both its potential benefits and possible risks. Several clinical studies have been carried out to determine the potential risks associated with the testosterone therapy to various organs including cardiovascular, respiratory, blood, prostate, and testes. Many of these studies were based on limited number of subjects and of short to medium duration. In spite of these limitations, these studies have identified several potential risks associated with the testosterone therapy. Some of the potential risks include possible effects on cardiovascular complications, sleep apnea, polycythemia, and prostate cancer.Elderly men seem to be at increased risk for fluid retention, increased risk of polycythemia, changes in sleep apnea, and acceleration of benign or malignant prostatic disease [5]. Testosterone-replacement therapy is not recommended for those who still desire fertility. A brief review of some of the main concerns associated with testosterone replacement therapy is given below.

### 7.1. Cardiovascular Disease

In the near past, there was a prevailing idea that androgens can have an atherogenic effect. This concern arose from the observation that aging men had higher incidence of CVD compared to aging women. Androgen administration drew a concern because it could subsequently add to the risk of developing CVD in men because there were already higher levels of testosterone in aging men compared to aging women. Over the last decade several papers have examined the relationship of androgens with CVD. Many of these studies suggest that there may be even neutral to beneficial effect of testosterone-replacement therapy on the cardiovascular risk factors and adverse cardiovascular complications (e.g., angina) [142, 176–179]. Testosterone replacement is not without risks for the aging males. TRT can lead to water retention and edema [176]. However, in one interventional trial, there were an increased number of cardiovascular-related adverse events in a population of older men who had significant chronic disease and with limitations with mobility [180]. However, such an increase was not noted in another study on testosterone treatment in frail older men [67]. We need longitudinal studies that will assess coronary artery changes to help us attain a better understanding of the effects of testosterone-replacement therapy on the cardiovascular system in aging hypogonadal men.

### 7.2. Sleep Apnea

Obstructive sleep apnea syndrome (OSA) can result in hypoxemia, sleep fragmentation, and excessive daytime sleepiness [181, 182]. Individuals with OSA can have an increased incidence of visceral obesity, insulin resistance, hypertension, and cardiovascular complications such as atrial fibrillation, stroke, and cardiac ischemia [181, 183]. OSA and metabolic syndrome are also associated with increased incidence of reduced-circulating testosterone values and erectile dysfunction [184–186]. Androgens may play role in contributing to the pathogenesis of obstructive sleep apnea as there are associations between men with abnormal sleep patterns and increased visceral adiposity with low plasma testosterone values [187, 188]. The presence of low testosterone levels with preexisting OSA can further complicate cardiometabolic risk factors.

 Some early studies have suggested supraphysiologic doses of testosterone seem to be more often associated with exacerbation of OSA [189–191]. Elderly individuals who are not treated for OSA should not be started on TRT till OSA is treated. This is because of the increased risk of polycythemia and its possible effects on other comorbid conditions.

Individuals with treated OSA and hypogonadism have been considered for testosterone-replacement therapy. In a meta-analysis of placebo-controlled trials of testosterone-replacement therapy to aging men, they found that there was no significant difference in frequency of sleep apnea between the placebo and testosterone-replacement therapy groups [101]. A small study has noted that testosterone-replacement therapy in individuals with idiopathic hypogonadotropic hypogonadism improves slow wave sleep and does not increase the frequency of OSA [192]. Another study noted that testosterone replacement therapy appears to improve erectile dysfunction in men with OSA and hypogonadism [187]. Even though small studies have shown benefits of testosterone-replacement therapy for individuals with OSA, one should exercise caution in giving testosterone-replacement therapy to individuals with severe untreated or poorly treated OSA [27, 101, 191]. Further longitudinal studies are needed to ascertain the effects of testosterone-replacement therapy in men with hypogonadism and OSA.

### 7.3. Polycythemia

Men with hypogonadism have lower hemoglobin levels than age-matched controls. The anemia, observed in the aging hypogonadal men, has been suggested to be partly due to declining testosterone levels and also partly due to effects on erythropoietin and erythroid progenitor cells [193–196]. Testosterone-replacement therapy can restore the hemoglobin levels of older men to the normal range. There appears to be a direct relation between testosterone dosage and the incidence of erythrocytosis with testosterone gel [157]. This dose-dependency was noted in another study [57], which compared the effects of transdermal versus intramuscular testosterone. It was noted that the intramuscular testosterone raised the hematocrit more than transdermal testosterone. The dose dependency was also seen in testosterone pellets [197]. The stimulation of hematopoiesis has been noted to be influenced by age and appears to be more pronounced in older men [198, 199].

Although an increase in the hematocrit is generally beneficial for hypogonadal men with anemia, an elevation of hematocrit above the normal range may lead to an increase in blood viscosity. The elevations in hemoglobin can result in adverse outcomes, particularly in elderly due to increases in viscosity that can exacerbate vascular disease (coronary, cerebrovascular, or peripheral vascular circulation) [27, 193, 200–202]. Individuals with increased blood viscosity have been known to be at an increased risk for thrombotic complications such as stroke, myocardial infarction, deep vein thrombosis, stroke, and pulmonary embolism [203–208]. This finding has raised awareness of the importance of monitoring hematocrit when on testosterone-replacement therapy. While there are legitimate concerns regarding polycythemia in individuals with testosterone supplementation, there is no clear evidence of significant complications in a recent meta-analysis of placebo-controlled trials [101].

The risk of polycythemia can be managed through careful monitoring of individuals on testosterone treatment [27, 180, 194, 209]. Individuals who are on testosterone-replacement therapy need a check of their hematocrit levels every 3 to 6 months and then annually [27]. The testosterone-replacement therapy should be held if the hematocrit is ≥54% until the hematocrit can return to a safe level; subsequently testosterone-replacement therapy is reinitiated at a reduced dose [27]. It is recommended that individuals with baseline hematocrit values (pre initiation of treatment with testosterone replacement therapy) above 50% should undergo a workup prior to testosterone-replacement therapy because these men have an increased chance of developing hematocrit levels above 54% [27].

### 7.4. Prostate

 There are reports of metastatic prostate cancer after testosterone administration in (elderly) men [210–212]. This has raised concern that testosterone-replacement therapy should be given to aging men who do not have significantly high risk of developing prostate cancer. The current Endocrine Society Guidelines have been developed to render testosterone administration to elderly men acceptably safe therapy in men without a prior history of prostate carcinoma or without evidence of harboring a prostate carcinoma [27].

 The concern for prostate cancer has led to the relative contraindication of testosterone-replacement therapy in some individuals [27]. If an individual's medical history reveals an increased risk of prostate cancer (e.g., African-Americans or men with first-degree relatives with prostate cancer) with PSA levels that are greater than 3 ng/mL, then the subject should have a workup with an urologist or other physician experienced in prostate gland evaluation. A workup with an urologist is also warranted in individuals with palpable prostate nodule or induration. Caution should be exercised in men over 40 years of age with increase in PSA concentration greater than 1.4 ng/mL within a 12-month period, elevated PSA levels (>3 or >4 ng/mL), or with high-risk prostate history. These individuals need to be evaluated with a digital rectal examination of the prostate to look for nodules and enlargement [27].

While there are relative contraindications to testosterone-replacement therapy, most studies (using different testosterone formulations over periods ranging from several months to 15 years in men with a wide range of ages) have not revealed an increased risk of prostate cancer [148, 213–215]. A meta-analysis found that testosterone treatment in older men compared to placebo was not associated with a significantly higher risk of prostate cancer [101]. Two small studies have reported no significant prostate-specific antigen (PSA) rise or prostate cancer recurrence in a total of 17 men, following radical prostatectomy in men with undetectable PSA [216, 217]. Another small study reported no cancer recurrence in 31 hypogonadal men treated with brachytherapy with a followup of approximately 5 years [218].

### 7.5. Other Potential Adverse Effects of Testosterone-Replacement Therapy

Due to an imbalance in the testosterone to estrogen ratio, hypogonadal men have reported experiencing softer testes, gynecomastia, and increased visceral obesity. Testosterone-replacement therapy does appear to be helpful in men to reduce some of these effects such reduced visceral obesity [107]. However, some men have reported adverse physical changes in their breast, testes, and skin after receiving testosterone-replacement therapy [5].

The increase in serum testosterone levels can suppress gonadotrophin release from the pituitary. The reduction in the production of intratesticular testosterone can also lead to reduced sperm production. Men should be advised that fertility can be adversely affected during testosterone-replacement therapy [219]. When men become hypogonadal, there is an alteration in the free estrogen to free androgen ratio and this can affect the breast [220]. Some men have noted experiencing breast tenderness and swelling or worsening breast tenderness in men with preexisting gynecomastia. The evidence for this association, however, is considered to be weak.

A change in testosterone levels through the use of testosterone replacement therapy appears to affect the skin and hair in men. Some men have noted increased oiliness of skin and acne. The increases in testosterone levels following testosterone-replacement therapy can increase secretion of sebum [221, 222]. This can increase the incidence of minor inconveniences such as the reappearance of acne and oiliness of skin. While there are anecdotal reports of changes in hair pattern, it has not been extensively reported in randomized double blind control trials. The transdermal formulations of testosterone replacement therapy can have administration specific skin reactions. Individuals who have used gels and patches have complained of skin irritation. However, there are more complaints of skin irritations (e.g., erythema, pruritus) that have been reported with patches than with gel preparations [223]. Intramuscular injections of testosterone can cause local reactions such as soreness, erythema, and bruising [224, 225]. The adverse affects of different formulations are listed in Table 3.

## 8. Summary and Recommendations

Longitudinal studies have shown that prevalence of hypogonadism in the aging men is increasing with each additional decade. Hypogonadism in older men is a syndrome characterized by low serum testosterone levels and clinical symptoms often seen in hypogonadal men of younger age. The patient is likely to be hypogonadal if the early morning serum total testosterone level is less than 250 ng/dL. Since the serum testosterone threshold for a given symptom may vary among symptoms and individuals, it is possible that this threshold is too low in some cases. More large scale data are required to clarify this uncertainty. Aging men with hypogonadism may experience many symptoms including decreased sexual function, decreased cognitive function, elevated LDL in the lipid profile, increased visceral adiposity, changes to the bone density and strength, and muscle weakness and atrophy. Late-onset hypogonadism may also have effects on diabetes and the cardiovascular system. The Endocrine Society Guidelines recommend that one should have symptoms of androgen deficiency and low testosterone levels for the diagnosis of hypogonadism. These guidelines also recommend that testosterone levels should be measured on more than one occasion and the samples for analysis need to be obtained in the morning before 10 am. The diagnosis of late-onset hypogonadism can be less certain in aging men who have comorbid conditions with borderline low testosterone levels.

 Several testosterone formulations have been developed for treatment of hypogonadism, and these formulations include testosterone injections, transdermal patches, oral testosterone, buccal testosterone, and transdermal testosterone gel preparations. Currently, testosterone injections and testosterone gel preparations are more commonly used in the United States. Limited clinical trials carried out with these medications indicate that testosterone-replacement therapy provides significant improvements in symptoms for men with late-onset hypogonadism. The long-term benefits and risks of testosterone-replacement therapy will become clearer when the effects of testosterone are studied on all health-related outcomes over an extended period of time. A NIH-sponsored large multicenter randomized control trial of testosterone in aging men with low testosterone levels is currently underway. This trial may provide answers to potential benefits and risks of testosterone replacement in aging males. One limitation of this trial is that it is not powered to fully assess potential risks of prostate cancer and cardiovascular events.

If an aging male is diagnosed with late-onset hypogonadism, the health care provider should engage in a discussion regarding the benefits and potential risks of testosterone therapy in older men. Older men who have significant erythrocytosis, untreated sleep apnea, prostate cancer, and high risk of cardiovascular events should be excluded from testosterone-replacement therapy. Currently, there is not enough evidence to clearly state that the benefits of testosterone-replacement therapy outweighs the risks of testosterone-replacement therapy in aging males. One cannot make a recommendation that testosterone-replacement therapy can be given to all aging males with low testosterone levels independent of significant signs or symptoms.

## Figures and Tables

**Table 1:** Cross-Sectional and Longitudinal Studies of Hypogonadism in Aging Men.

Study	Population	Results	Notes
European Male Aging Study (Cross-sectional) [10]	3219 men ages 40 to 79 years.	(1) Overall prevalence of hypogonadism was 2.1%. (2) Hypogonadism increases with age 0.1% (40 to 49 yrs) 0.6% (50 to 59 yrs), 3.2% (60 to 69 yrs) 5.1% (70 to 79 yrs). (3) Prevalence is higher with increasing number of coexisting illnesses and BMI	Total testosterone <320 ng/dL (11 nmol/L), and free testosterone <64 pg/mL (220 pmol/l). (LCMS method)

The Baltimore Longitudinal Study of Aging (longitudinal) [8]	890 men; average age 53.8 + 16 (samples during time period 1961 to 1995).	(1) Serum testosterone decreased at a fairly constant rate, independent of other clinical variables. (2) Average change of T is about 3.2 ng/dL (−0.124 nmol/L) per yr. (3) Incidence of hypogonadism:: ~20% in 60s, ~30% in 70s, and ~50% in 80s.	Androgen deficiency was defined as total testosterone less than 325 ng/dL. (RIA method)

The Massachusetts Male Aging Study (longitudinal) [12]	1667 men aged 40 to 70 at baseline (1987–1989).	(1) Crude prevalence of androgen deficiency at baseline and followup is 6.0 and 12.3%. (2) Crude incidence rate of androgen deficiency was 12.3 per 1,000 P-Yr. (3) Prevalence and Incidence rate increased with age. (4) T declines associated with aging −10.1% decline in TT per decade −23.8% decline in FT per decade.	Total testosterone less than 200 ng/dL or total testosterone 200–400 ng/dL and free testosterone less than 8.91 ng/dL. (RIA method)

Boston Area Community Health Survey [9]	1475 men ages of 30–79 yr; 47.3 ± 12.5 yr.	(1) Crude prevalence of symptomatic androgen deficiency was 5.6%. (2) Prevalence increases with age a. 3.1–7.0% in men less than 70 yr b. 18.4% among 70 yr old. (3) 24% of subjects had total testosterone less than 300 ng/dL, (4) 11% of subjects had free testosterone less than 5 ng/dL	Total concentration <300 ng/dL and free testosterone <5 ng/dL.

New Mexico Aging Process Study (longitudinal) [19]	77 men in the age group 66–80. 15 years of the study period.	(1) Observed a longitudinal decline in T and an increase in LH and FSH. (2) The increasing levels of FSH suggest that hypogonadism in aging males is probably due to secondary hypogonadism. (3) Average rate of decrement in testosterone concentration is about 11 ng/dL (0.382 nmol/L) per year	Note, this study varies in rate of testosterone decline from the other studies

Abbreviations: T: Testosterone; TT: Total Testosterone; FT: free testosterone; YRS: years of age; P-Yr: person years; LCMS: Liquid Chromatography tandem Mass Spectrometry (LCMS); RIA: Radio Immunometric Assay.

**Table 2:** Symptoms and associated morbidities with low testosterone levels.

Symptoms and associated morbidities	

Sexual function	Cognition and vitality
Loss of libido	Decline in verbal and visual memory
Erections: reduced quality and frequency, including nocturnal erections	Decline in visuospatial performance
Oligospermia or azoospermia	Depressed mood
Gynecomastia/breast discomfort	Decreased energy
Changes in secondary hair characteristics (e.g., shaving)	Decline in feelings of initiative
Changes in size of testes	Decreased sense of vitality
Decreased fertility	

Muscle, bone, and body composition	Other

Progressive decrease in muscle mass	Sleep disturbance
Decreased physical function	Lipid abnormalities
Decrease in bone mineral density; osteopenia, osteoporosis, increased risk of bone fractures	Mild anemia (normochromic, normocytic)
Increase in visceral fat	Decreased response to PDE5 inhibitors

**Table 3:** Advantages and disadvantages of testosterone preparations.

Administration method	Formulation	Advantages	Disadvantages
Transdermal agents	Testosterone patches	Mimics circadian rhythm; simple administration	Skin irritation, occasional allergic contact dermatitis, daily administration
	Testosterone gel 1-2%	Easy to apply, readily absorbed into skin. Flexible-dose modifications, skin irritation less common, T levels maintained in normal range.	Transfer during intimate contact; direct contact with children and women should be avoided; skin irritation at the application site in a small number of men, daily administration
	Underarm testosterone gel	Skin irritation less common, T levels maintained in normal range	Transfer during intimate contact; direct contact with children and women should be avoided, daily administration

Subcutaneous agents	Implants	Implants are inserted every 16 to 24 weeks	Invasive procedure with risk of extrusion and infection

Intramuscular injections	Testosterone cypionate	Relatively low cost	Pain and redness at injection site; fluctuations in circulating T levels high risk of polycythemia;
	Testosterone enanthate	Relatively low cost	Pain and redness at injection site; fluctuations in circulating T levels, high risk of polycythemia
	Testosterone undecanoate	Less frequent administration, T levels maintained in normal range	Pain at intramuscular injection site

Buccal formulation agents	Buccal testosterone	Provides sustained release of T; through the buccal mucosa	Unpleasant taste, can stick to gums, gum pain, or tenderness, headache

Oral formulation agents	Methyltestosterone	Oral; modifiable dosage, relatively low cost	Potential hepatotoxicity, drug not in use, may adversely affect lipid profile, decreasing HDL, and increasing LDL
	Testosterone undecanoate	Oral; (approved in the Europe)	Variable clinical effects and testosterone levels must be taken with meals, nonaromatizable to estrogen, Underevaluation in the United States

Abbreviations: T: Testosterone; High Density Lipoprotein: HDL; Low Density Lipoprotein: LDL.
